# Association between decreased HDL levels and cognitive deficits in patients with bipolar disorder: a pilot study

**DOI:** 10.1186/s40345-019-0159-7

**Published:** 2019-11-25

**Authors:** Li Hui, Xiao Li Yin, Jie Chen, Xu Yuan Yin, Hong Liang Zhu, Jin Li, Guang Zhong Yin, Xiao Wen Xu, Xiao Nan Yang, Zheng Kang Qian, Cai Xia Jiang, Zhen Tang, Hai Bing Yang, Eric F. C. Cheung, Raymond C. K. Chan, Qiu Fang Jia

**Affiliations:** 1 Institute of Mental Health, Suzhou Guangji Hospital, The Affiliated Guangji Hospital of Soochow University, Soochow University, No. 11 Guangqian Road, Suzhou, 215137 Jiangsu People’s Republic of China; 2 Wenzhou Kangning Hospital, Wenzhou Medical University, Wenzhou, Zhejiang People’s Republic of China; 3 Neuropsychology and Applied Cognitive Neuroscience Laboratory, CAS Key Laboratory of Mental Health, Institute of Psychiatry, Beijing, People’s Republic of China; 4 Suzhou Center for Disease Control and Prevention, Suzhou, Jiangsu People’s Republic of China; 5 Castle Peak Hospital, Hong Kong, People’s Republic of China; 6 Department of Psychology, University of Chinese Academy of Sciences, Beijing, People’s Republic of China; 7 CAS Key Laboratory of Mental Health, Institute of Psychology, 16 Lincui Road, Beijing, 100101 People’s Republic of China

**Keywords:** High density lipoprotein, Bipolar disorder, Cognition, RBANS

## Abstract

**Background:**

Cognitive deficits are common in patients with bipolar disorder (BD). Abnormal high density lipoprotein (HDL) levels have been implicated in cognitive deficits associated with ageing and neurodegenerative disorders. The present study aimed to investigate serum HDL levels, cognitive deficits and their association in patients with BD.

**Methods:**

Thirty-seven patients with BD and 37 gender- and age-matched healthy controls (HCs) were recruited in a case–control study. Cognition was assessed using the Repeatable Battery for the Assessment of Neuropsychological Status (RBANS), and serum HDL levels were measured using enzymatic colourimetry.

**Results:**

There was no difference in serum HDL levels between patients with BD and HCs after adjusting for gender, age, education and body mass index (BMI). Cognitive test scores in patients with BD were significantly lower than those in HCs except for the visuospatial/constructional index after adjusting for confounding variables. Serum HDL levels were positively correlated with RBANS total score and language score in patients with BD. Stepwise multiple regression analysis showed that serum HDL levels were significantly correlated with RBANS total score and subscale scores on immediate memory and language in patients with BD after adjusting for confounding factors.

**Conclusions:**

Our findings suggest that patients with BD had poorer cognitive performance than HCs except for the visuospatial/constructional domain, and decreased serum HDL levels were correlated with cognitive deficits, especially in immediate memory and language domains in patients with BD.

## Background

Bipolar disorder (BD) is a severe and heritable psychiatric illness with a prevalence of approximately 1–2%. It is characterized by recurrent episodes of depression and mood elevation (Merikangas et al. 2011; Waraich et al. 2004). Although BD mainly involves mood disturbance, cognitive deficits are also frequently observed in patients with BD. Previous studies have reported that BD patients have deficits in executive function, memory and attention (Palazzo et al. 2017; Robinson et al. 2006). A recent study has also shown that first degree relatives of BD patients have poorer verbal learning, processing speed and working memory than healthy controls (HCs) (Calafiore et al. 2017). Therefore, cognitive deficits may be a potential endophenotype for BD, but the underlying pathophysiology of cognitive deficits in patients with BD is unclear.

High density lipoprotein (HDL) is a heterogeneous group of lipoprotein particles synthesized in the systemic circulation and the brain (Dietschy and Turley 2001; Hottman, et al. 2014; Segrest et al. 2000). Previous studies have found that reduction in HDL levels, which might influence the central nervous system (CNS), is a risk factor for affective disorders (Hottman et al. 2014; Sagud et al. 2009). A study has also reported that first degree relatives of BD patients have lower HDL levels than HCs, suggesting that decreased HDL may be a trait in patients with BD (Sobczak et al. 2002). Compared with HCs, patients with anxiety disorder have been found to have significantly reduced serum HDL levels (Peter et al. 2002). Moreover, HDL levels have been found to be negatively correlated with scores on the positive subscale of the Positive and Negative Syndrome Scale in smoking patients with schizophrenia (An et al. 2016). Therefore, abnormal serum HDL levels may be involved in the pathophysiology of BD.

A previous study has reviewed the effect of HDL levels on cognitive function in ageing and neurodegenerative disorders, and suggested that HDL-enhancing approaches may have the potential to improve cognitive performance (Hottman et al. 2014). Previous studies have reported that HDL levels may play a critical role in maintaining cognitive function during ageing (Atzmon et al. 2002; Barzilai et al. 2006; Song et al. 2012; van den Kommer et al. 2012). HDL levels have been found to be positively correlated with cognitive performance in patients with Alzheimer’s disease (Warren et al. 2012), while reduced HDL levels has also been found to be associated with cognitive deficits in patients with atherosclerotic disorder (van Exel et al. 2002). Moreover, a study has shown that HDL levels may influence cognitive performance in middle-aged adults (Singh-manoux et al. 2013). However, no study has investigated the relationship between HDL levels and cognitive deficits in patients with BD using standardized instruments such as the Repeatable Battery for the Assessment of Neuropsychological Status (RBANS). Therefore, the objectives of this study were to examine whether: (1) patients with BD have poorer cognitive performance than HCs; and (2) reduced serum HDL levels are associated with cognitive deficits in patients with BD.

## Materials and methods

### Ethics statement

The research protocol and informed consent were approved by the Institutional Ethics Committee of the Affiliated Guangji Hospital of Soochow University. All experiments were performed in accordance with the approved guidelines and regulations. All participants provided written informed consent.

### Participants

Thirty-seven patients with BD (male/female = 15/22) were recruited from the Affiliated Guangji Hospital of Soochow University, Suzhou City, China. The following inclusion criteria were applied: (1) 18–50 years of age; (2) Han Chinese descent; (3) a diagnosis of BD according to the Diagnostic and Statistical Manual of Mental Disorders, Version Four (DSM-IV) (First et al. 1996); and (4) at least 5 years of education and able to participate in cognitive assessment. Some of the participants were prescribed with valproate or lithium, while some were prescribed with antipsychotic medications. Doses of antipsychotic medications were converted into chlorpromazine equivalence (Kane et al. 1998; Woods 2003). The diagnosis of the participants was ascertained by two experienced psychiatrists using the Structured Clinical Interview for DSM-IV (First et al. 1996). The severity of depressive and manic symptoms was assessed using the Hamilton Depression Rating Scale (HAMD) (Hamilton 1960) and the Bech–Rafaelsen Mania Rating Scale (BRMS) (Bech et al. 1979).

Thirty-seven gender- and age-matched HCs (male/female = 15/22) were recruited from the local community in the Gusu District of Suzhou. Current mental status and personal or family history of mental disorders were assessed using unstructured interviews. None of the HCs had a personal or family history of psychiatric disorders.

All participants were in good physical health. Moreover, potential participants were excluded if they met any of the following exclusion criteria: (1) a personal or family history of neurodegenerative disorders, anxiety disorder, major depressive disorder, schizophrenia, or schizoaffective disorder; (2) a history of cardiovascular disease, cerebrovascular disease, cancer, diabetes, hypertension and pregnancy; and (3) a history of drug or alcohol abuse/dependence.

### Clinical and neuropsychological assessment

Information including age, gender, education, body mass index (BMI), age of illness onset, duration of illness, number of episodes, number of hospitalizations, medication details and clinical symptoms was obtained from each participant, supplemented by review of medical records.

The RBANS (Form A) is a brief standardized tool which provides a general evaluation of several cognitive domains (Randolph et al. 1998). The RBANS includes 12 sub-tests that generate five index scores and a total score. The five indices include immediate memory (consisting of list learning and story memory tests), visuospatial/constructional domain (consisting of figure copying and line orientation tests), language (consisting of picture naming and semantic fluency tests), attention (consisting of digit span and coding tests) and delayed memory (consisting of list recall, story recall, figure recall and list recognition tests). The RBANS has been translated into Chinese, and its validity and test–retest reliability have been established in patients with schizophrenia and normal volunteers (Zhang et al. 2008). To ensure consistency and reliability of rating, two psychiatrists simultaneously attended a training session for standardizing the use of RBANS before the start of this study. The intraclass correlation coefficient was 0.94.

### HDL measurement

Blood samples from patients with BD and HCs were collected between 7 and 9 a.m. following an overnight fast to limit possible bias. The blood samples were then allowed to clot at room temperature and centrifuged at 3500 rpm for 4 min. Serum samples were separated, aliquoted and stored at − 80 °C in a refrigerator before use. Serum HDL levels were measured using enzymatic colourimetry with a Hitachi automatic biochemistry analyzer (cobas 8000 ISE 900 Module, Hitachi High-Technologies Corporation, Japan) and commercially available kits (HDL-Cholesterol plus 3rd generation, Medical System Biotechnology, Ningbo, China). The sensitivity of this kit was 0.08 mmol/l, and its intra- and inter-assay variation coefficients were 1.13% and 1.00%, respectively. Serum samples were assayed by a technician blind to the clinical status of all participants and each assay was run in duplicate.

### Data analysis

Clinical variables, RBANS scores and serum HDL levels between patients with BD and HCs were compared using analysis of variance (ANOVA) for continuous variables and Chi-square test for categorical variables. If there were significant differences in RBANS scores and serum HDL levels between the two groups, analysis of covariance (ANCOVA) was carried out with RBANS scores and serum HDL levels as independent variables, and age, gender, education and BMI as covariates. The correlation between serum HDL levels and RBANS scores in patients with BD and HCs was calculated using Pearson’s product moment correlation coefficients. Further stepwise multivariate regression analysis was used to evaluate the correlations between serum HDL levels and RBANS scores after adjusting for various confounding variables in the two groups. SPSS version 22.0 was used for all statistical analysis. Statistical significance was set at *P *< 0.05.

## Results

### Sample characteristics

The clinical data of the participants are shown in Table 1. There was no difference in gender, age, education and BMI between patients with BD and HCs (all, *P *> 0.05). The mean and standard deviation (mean ± SD) of age of illness onset (years), duration of illness (months), number of episodes, and number of hospitalizations in patients with BD were 21.41 ± 7.62, 107.62 ± 101.94, 4.84 ± 1.48, and 3.08 ± 2.88, respectively. Thirty patients with BD were prescribed with valproate (81.10%), three with lithium (8.10%), and four were not taking any mood stabilizers (10.80%). The mean antipsychotic dosage of the participants in chlorpromazine equivalence was 189.13 ± 222.19 mg/day.

**Table 1:** Demographic and clinical characteristics in patients with BD and HCs

	Patients with BD (n = 37)	HCs (n = 37)	*χ*^*2*^ or *F*	*P*
Gender (male/female)	15/22	15/22	0.00	1.00
Age (years)	29.78 ± 10.05	29.95 ± 9.02	0.01	0.94
Education (years)	10.14 ± 2.98	11.35 ± 2.36	3.78	0.06
BMI (kg/m^2^)	22.94 ± 3.97	21.86 ± 2.84	1.80	0.18
Age of illness onset (years)	21.41 ± 7.62			
Duration of illness (months)	107.62 ± 101.94			
Number of episodes	4.84 ± 1.48			
Number of hospitalizations	3.08 ± 2.88			
Chlorpromazine equivalents (mg/day)	189.13 ± 222.19			
BRMS	7.04 ± 6.88 (range 0–4)			
HAMD	13.97 ± 10.24 (range 0–4)			
Frequently used MS				
Valproate	30 (81.10%)			
Lithium	3 (8.10%)			
Not taking MS	4 (10.80%)			

*BD* bipolar disorder, *HCs* healthy controls, *BMI* body mass index, *BRMS* Bech–Rafaelsen Mania Rating Scale, *HAMD* Hamilton Depression Rating Scale, *MS* mood stabilizers

### Comparison of serum HDL levels between patients with BD and HCs

**Fig. 1:**
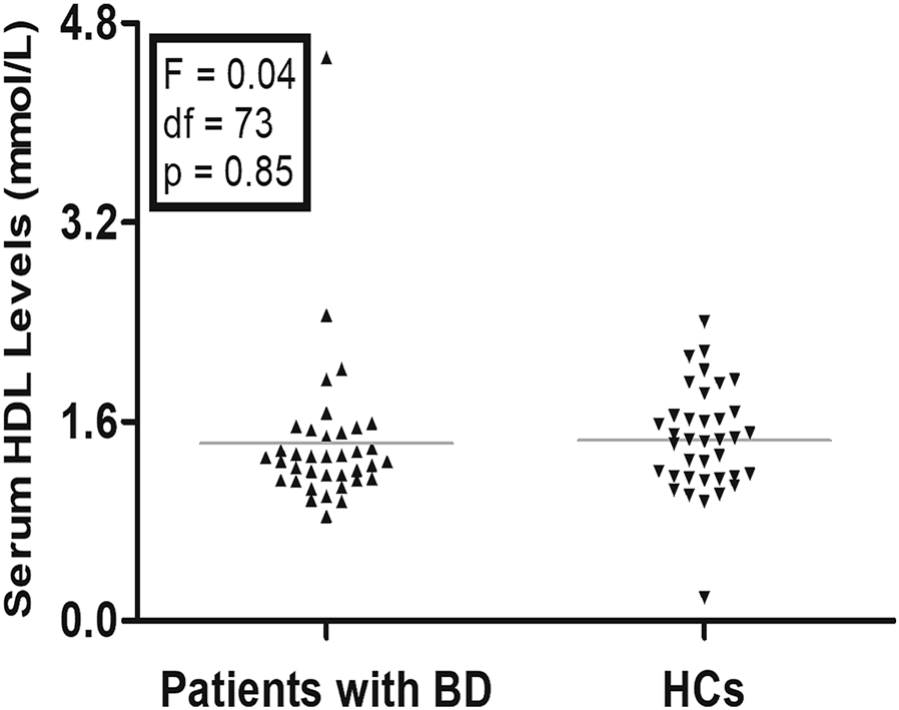
There was no difference in serum HDL levels between patients with BD and HCs (1.43 ± 0.61 mmol/l vs. 1.45 ± 0.42 mmol/l, F = 0.04, df = 73, p = 0.85)

The mean ± SD of serum HDL levels was 1.43 ± 0.61 mmol/l in patients with BD and 1.45 ± 0.42 mmol/l in HCs (Fig. 1). There was no difference in serum HDL levels between patients with BD and HCs (F = 0.04, df = 73, *P *= 0.85). The results remained unchanged after adjusting for gender, age, education and BMI (*P *> 0.05).

### Comparison of RBANS scores between patients with BD and HCs

The mean ± SD of RBANS scores in patients with BD and HCs are shown in Table 2. There were significant differences in cognitive function including RBANS total score (F = 19.08, *P *< 0.001) and subscale scores on immediate memory (F = 8.34, *P *= 0.005), language (F = 4.87, p = 0.03), attention (F = 21.17, *P *< 0.001), and delayed memory (F = 18.31, *P *< 0.001) between the two groups except for the visuospatial/constructional domain (F = 1.90, *P* = 1.70). These differences remained significant after adjusting for gender, age, education, BMI and serum HDL levels (all, *P *< 0.05).

**Table 2:** Comparison of the RABNS total and index scores between patients with BD and HCs

Index	Patients with BD (n = 37)	HCs (n = 37)	*F*	*P*	Adjusted *F*	Adjusted *P*^a^
Immediate Memory	75.57 ± 12.62	84.84 ± 14.90	8.34	0.005	5.05	0.03
Visuospatial/Constructional	74.59 ± 14.02	78.86 ± 12.56	1.90	0.17	0.74	0.39
Language	85.70 ± 16.32	93.19 ± 12.62	4.87	0.03	5.29	0.03
Attention	89.89 ± 16.09	106.73 ± 15.39	21.17	< 0.001	16.08	< 0.001
Delayed memory	75.41 ± 15.58	87.95 ± 8.67	18.31	< 0.001	16.24	< 0.001
Total score	74.73 ± 12.37	86.59 ± 10.96	19.08	< 0.001	17.43	< 0.001

^a^Adjusted *F* indicated the *F* value after controlling for gender, age, education, BMI and serum HDL levels

### Correlation of serum HDL levels with cognitive performance

**Fig. 2:**
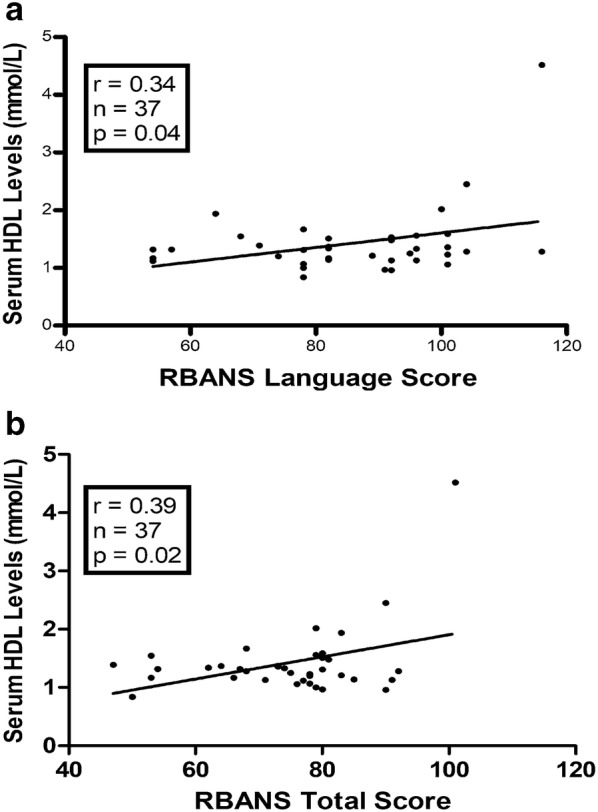
For patients with BD, **a** serum HDL levels were positively associated with the language score (r = 0.34, P = 0.04); **b** there was the positively correlation of serum HDL levels with the RBANS total score (r = 0.39, P = 0.02)

In patients with BD, correlation analysis showed that serum HDL levels were significantly correlated with RBANS total score (r = 0.39, *P *=0.02) and subscale score on language (r = 0.34, *P *=0.04) (Fig. 2). However, there were no significant correlations between serum HDL levels and RBANS total score and subscale scores on immediate memory, attention, visuospatial/constructional domain, language, and delayed memory in HCs (all, *P *> 0.05).

Further stepwise multivariate regression analysis showed that serum HDL levels were significantly correlated with RBANS total score (*β *= 8.63, t = 3.00, *P *= 0.006) and subscale scores on immediate memory (*β *= 6.92, t = 2.14, *P *= 0.04), and language (*β *= 8.35, t = 2.09, *P *= 0.04) in patients with BD after adjusting for age, gender, education, BMI, age of illness onset, duration of illness, number of episodes, number of hospitalizations, medication dosage and clinical symptoms, but not in HCs (all, *P *> 0.05).

## Discussion

To the best of our knowledge, this is the first study that investigates the relationship between serum HDL levels and cognitive deficits in patients with BD. We found that BD patients had poorer cognitive function except for the visuospatial/constructional domain compared with HCs, and serum HDL levels were significantly correlated with cognitive deficits, especially in immediate memory and language in patients with BD.

Significant differences in HDL levels between first degree relatives of BD patients and HCs have been reported, suggesting that reduced HDL levels may be a potential biomarker for BD (Sobczak et al. 2002). Moreover, serum HDL levels have been found to be negatively correlated with clinical symptoms in patients with some psychiatric disorders (An et al. 2016). However, we found that there was no difference in serum HDL levels between patients with BD and HCs. Interestingly, several studies have found that reduced HDL levels may be associated with cognitive deficits (Hottman et al. 2014; Sagud et al. 2009; Zuliani et al. 2010). We also found that patients with BD had more severe cognitive deficits than HCs except for the visuospatial/constructional domain. This finding is consistent with several previous studies in BD patients (Dickerson et al. 2004, 2011; Gerber et al. 2012). However, other studies have reported different findings. For example, a recent study found deficits in visuospatial/constructional function, but not deficits in immediate memory, attention and delayed memory in Northern Chinese individuals with BD (Bo et al. 2018). Another study also reported that patients with BD appear to have normal attention and delayed memory in an Australian population (Gogos et al. 2010). Moreover, a previous study has found that patients with BD exhibit normal immediate memory and delayed memory in an American population (Dittmann et al. 2007). These inconsistent findings of cognitive deficits in patients with BD could be due to multiple factors such as differences in genetic background, gender, age, education, smoking status, illness duration and exposure to treatment. Therefore, the underlying neurobiological mechanisms of cognitive deficits in patients with BD are still not fully understood and warrant further investigation in the future.

We also found that serum HDL levels were positively correlated with cognitive function in patients with BD, which suggests that abnormal serum HDL levels may influence cognitive function in patients with BD. Several studies have found that HDL levels are positively correlated with cognitive function, which could be improved by enhancing HDL levels in ageing individuals and patients with neurodegenerative disorders (Atzmon et al. 2002; Barzilai et al. 2006; Hottman et al. 2014; Song et al. 2012; Warren et al. 2012; van den Kommer et al. 2012). The major protein component of HDL is apoA-I, which is responsible for most HDL functions (Segrest et al. 2000). The mechanism underlying the effect of HDL level on cognitive function could be related to the role of apoA-I/LDH in neuroprotection (Cockerill et al. 1995; Navab et al. 2000). This hypothesis is supported by several previous studies. For example, the reconstituted human apoA-I-containing HDL has been shown to be involved in the decline in neuronal impairment via anti-oxidative mechanism in stroke mice (Paternò et al. 2004). In addition, abnormalities in apoA-I protein levels and gene expression have been reported to influence the immune system of model animals and have been shown to be significantly correlated with cognitive performance (Buga et al. 2006; Handattu et al. 2009; Lewis et al. 2010; Saito et al. 1997). Thus, reduced serum HDL levels may result in impairment in anti-inflammatory and anti-oxidation properties, and may lead to cognitive deficits in patients with BD.

This study has several limitations. First, the sample size was relatively small. Thus, our results should be regarded as preliminary and future studies with a large sample size are warranted. Second, this study was a cross-sectional study and could not determine the causality between decreased serum HDL levels and cognitive deficits in patients with BD. Future longitudinal studies are needed to clarify their causality. Third, HDL levels only reflect changes in one lipoprotein. Future studies should consider studying a network of lipoproteins. Finally, clinical information which might confound our results, including smoking status, sleep status and fatigue, were not collected and might have biased our findings.

## Conclusions

In conclusion, our results suggest that BD patients had poorer cognitive performance than HCs except for the visuospatial/constructional domain, and reduced serum HDL levels may be correlated with cognitive deficits in patients with BD.

## Data Availability

The data that support findings of this study are available from the corresponding author (L.H.). The data are not publicly available due to in the need to safeguard the privacy of the participants.
